# Boards of Examiners

**Published:** 1897-08

**Authors:** 


### BOARDS OF EXAMINERS.

---

The Virginia State Board of Veterinary Examiners held examinations on the 23d and 24th of June and on July 8, three candidates passing successful examinations, as follows: Dr. B. A. Brown, of Ontario and National Veterinary Colleges; Dr. A. M. Lushington (colored), of the Veterinary Department of the University of Pennsylvania, and Dr. S. C. Simpson, of Ontario College. The board will hold its next regular meeting in Richmond, Va., January 4, 1898.

Dr. David W. Cochran (late lecturer on horseshoeing, American Veterinary College, and a practical horseshoer) with Dr. Quinn (State Examiner of Horseshoers for New York State) were the examiners of students lectured to during the past winter by Drs. Huidekoper and Gill, and the result was very gratifying. Five men received 80 per cent. or better; two men received 70 per cent.; two men received 60 per cent., and two men received 50 per cent. A gold medal was offered by Mr. Goldsticker to the one passing the best examination, and was awarded to Mr. P. B. McSwegan.

**What will the Chicagoans present for the program?**

Some eight veterinarians entered for examination for appointment in the Bureau of Animal Industry at the civil service examination at Boston on April 26, 1897.

Drs. S. J. Buckley and B. A. Brown successfully passed the examination of the Maryland Board. One applicant failed to pass.

